# Insights into the Virulence and Antimicrobial Resistance of *Staphylococcus hyicus* Isolates from Spanish Swine Farms

**DOI:** 10.3390/antibiotics13090871

**Published:** 2024-09-11

**Authors:** Oscar Mencía-Ares, Eva Ramos-Calvo, Alba González-Fernández, Álvaro Aguarón-Turrientes, Ana Isabel Pastor-Calonge, Rubén Miguélez-Pérez, César B. Gutiérrez-Martín, Sonia Martínez-Martínez

**Affiliations:** 1 Department of Animal Health, Faculty of Veterinary, Universidad de León, 24007 León, Spain; eramoc00@estudiantes.unileon.es (E.R.-C.); algonf@unileon.es (A.G.-F.); anaipastor00@gmail.com (A.I.P.-C.); rmigp@unileon.es (R.M.-P.); cbgutm@unileon.es (C.B.G.-M.); smarm@unileon.es (S.M.-M.); 2 Laboratorios SYVA, 24009 León, Spain; alvaro.aguaron@syva.es

**Keywords:** antimicrobial resistance, biofilm formation, exfoliative toxins, exudative epidermitis, *Staphylococcus hyicus*, virulence

## Abstract

*Staphylococcus hyicus* is a significant pathogen in swine, primarily causing exudative epidermitis. Addressing *S. hyicus* infections requires both the characterization of virulence and antimicrobial resistance (AMR) in farm-recovered isolates. This study aimed to characterize the virulence, AMR, and biofilm formation of *S. hyicus* isolates from Spanish swine farms. A total of 49 isolates were analyzed, originating from animals with cutaneous, reproductive, and systemic clinical signs. Half of the isolates (49.0%) were positive for at least one virulence factor (VF) gene, with *SHETA* being the most frequent (28.6%). A high frequency of multidrug resistant (MDR) isolates was observed (83.7%), with significant resistance to commonly used antimicrobials, including lincosamides (83.7%), pleuromutilins (81.6%), penicillins (75.5%), and tetracyclines (73.5%). All isolates exhibited robust in vitro biofilm formation capacity (DC = 15.6 ± 7.0). Significant associations were found between VFs, biofilm formation, and AMR patterns, highlighting the link between the resistance to lincosamides and pleuromutilins (*p* < 0.001; Φ = 0.57) and macrolides (*p* < 0.001; Φ = 0.48), and the association of AMR with the *ExhC* and *ExhD* VF genes. These findings underscore the need for targeted diagnostics to improve management and therapeutic strategies to mitigate the impact of *S. hyicus* on swine production.

## 1. Introduction

*Staphylococcus hyicus*, a zoonotic pathogen that very rarely causes bacteriemia and sepsis in humans [1], is the causative agent of porcine exudative epidermitis (EE) in pigs, primarily affecting suckling and newly weaned piglets [2]. EE is characterized by a generalized or located skin disorder, including exfoliation, sebaceous secretion, and the formation of a brownish coat of exudate that may cover the entire body [3]. Additionally, *S. hyicus* has been associated with other clinical conditions such as sow mastitis and metritis [4], as well as systemic infections, including arthritis, in weaning piglets [5].

*S. hyicus* can be categorized into toxigenic and non-toxigenic strains based on their ability to cause EE in pigs [6]. The pathogenicity of toxigenic *S. hyicus* is mainly determined by exfoliative toxins, which are the primary virulence factors (VFs) inducing EE [7]. To date, six exfoliative toxins have been identified, including ExhA, ExhB, ExhC, and ExhD, primarily described in Denmark [8], and SHETA and SHETB, firstly described in Japan [9,10]. Another relevant VF that may be considered is biofilm formation. Although no specific information is available on biofilm formation by *S. hyicus*, other *Staphylococcus* species are known to possess a considerable number of genes encoding for biofilm production, adhesion factors, and exoenzymes [11]. These structured aggregates of bacterial cells surrounded by an extracellular matrix enhance bacterial survival by impairing both the host immune system and the effectiveness of antimicrobials [12], leading to therapeutic failure.

Addressing clinical conditions caused by *S. hyicus* requires both the characterization of the virulence of farm-recovered isolates and the evaluation of their antimicrobial resistance (AMR). Accurate AMR characterization is crucial for guiding effective antimicrobial therapy and ensuring antimicrobial stewardship, as it contributes to limiting the development and spread of AMR in food-producing animals, which poses a significant risk to animal, environmental, and public health. This underscores the need for implementing more restrictive legislation on antimicrobial use in food-producing animals, such as that adopted by the European Union, particularly in relation to the restriction of prophylactic and metaphylactic use [13]. However, updated information on AMR in *S. hyicus* is sparse and largely limited to non-European regions [2,14], with no recent studies from Spain. Therefore, to expand and update the characterization of *S. hyicus*, this study aimed to characterize the virulence and antimicrobial susceptibility profiles of a selection of *S. hyicus* isolates recovered from Spanish pig farms, together with the first description of *S. hyicus* in vitro biofilm formation ability.

## 2. Results

### 2.1. Isolation and Characterization of Staphylococcus hyicus

A selection of 49 *S. hyicus* isolates were recovered from Spanish swine farms across ten regions from May to December 2023. A detailed summary of these isolates is available in Table 1. Most of the isolates originated from the skin (51.0%) and from pigs showing clinical signs compatible with epidermitis (57.1%). Notably, approximately a quarter of the isolates were from reproductive exudates (26.5%) and cases of metritis in sows (24.5%). Additionally, another quarter of the isolates had a visceral origin (joint, 14.3%; brain, 8.2%) from piglets with systemic clinical signs (18.4%) such as polyserositis. Furthermore, the isolates were not limited to clinical cases in suckling or weaning piglets (73.5%), but also included those from adult animals, particularly sows (26.5%). Finally, the isolates were obtained from both white crossbred (44.9%) and Iberian pigs (55.1%), indicating a broad distribution across two different breeds.

### 2.2. Characterization of Virulence Factors

**Figure 1:**
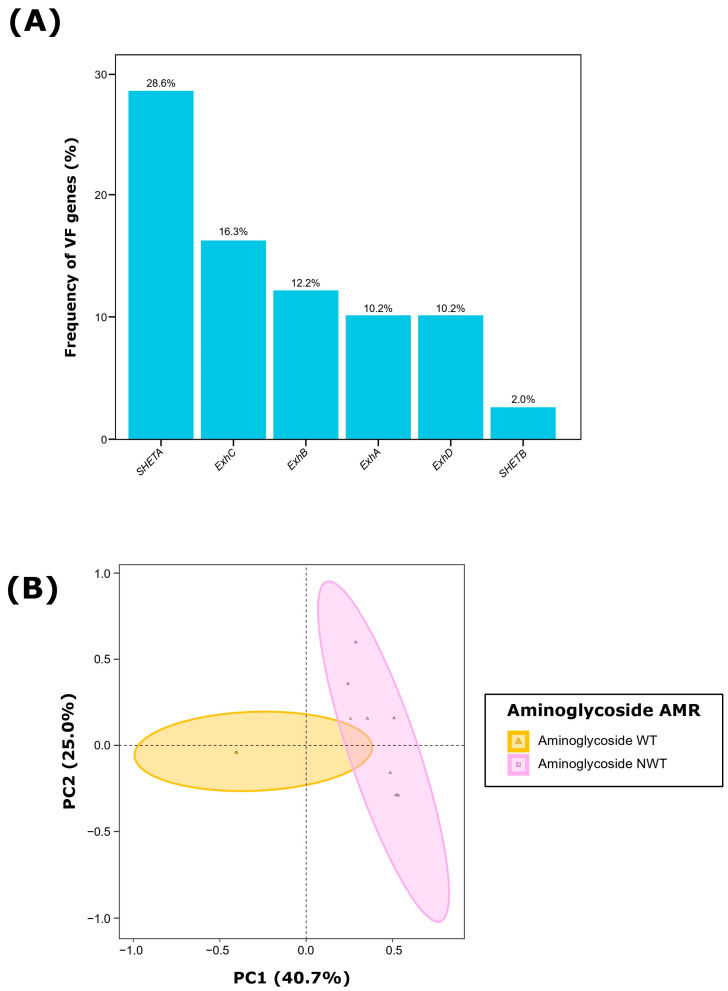
Virulence factor (VF) characterization of 49 *Staphylococcus hyicus* isolates from Spanish swine farms. (**A**) Frequency (%) of each VF gene and (**B**) Principal component analysis (PCA) of the six evaluated VF genes, showing grouping based on aminoglycoside antimicrobial resistance (AMR) of each *S. hyicus* isolate. WT: wild type, susceptible; NWT: non-wild type, resistant.

**Figure 2:**
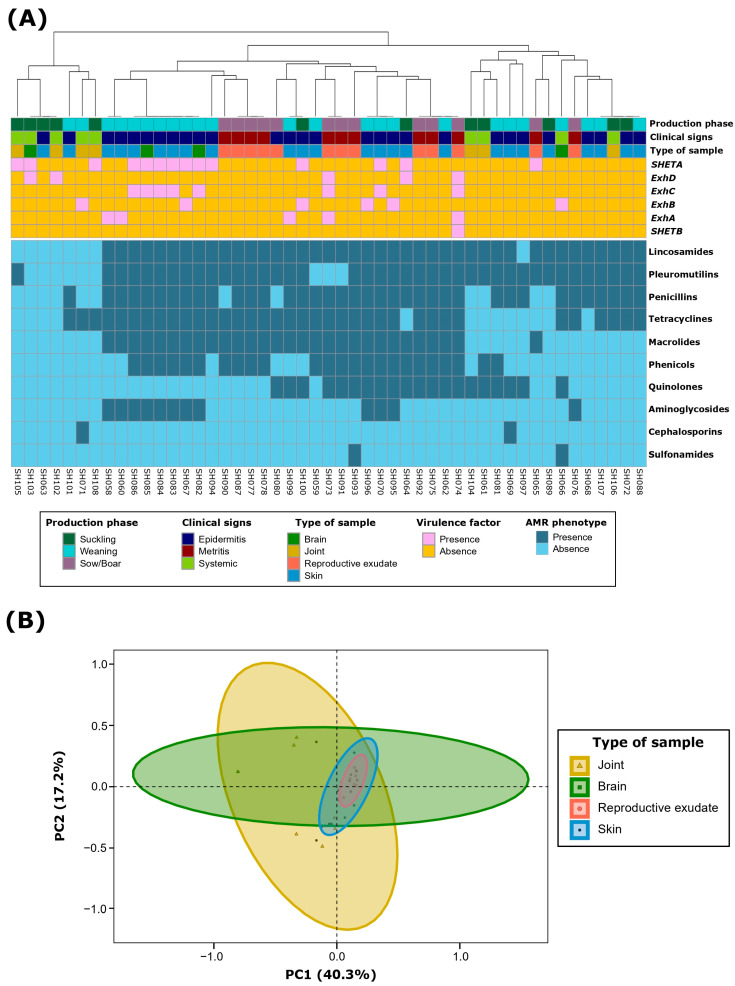
Antimicrobial resistance (AMR) characterization at the class level of 49 *Staphylococcus hyicus* isolates from Spanish swine farms. (**A**) AMR phenotype clustering based on antimicrobial classes, using the unweighted pair group method with arithmetic mean (UPGMA) as the hierarchical clustering method. (**B**) Principal component analysis (PCA) of the AMR patterns, showing grouping based on the type of sample of each *S. hyicus* isolate.

Among the 49 *S. hyicus* isolates, 49.0% (n = 24) harbored at least one of the VFs evaluated. The most frequently detected VF was *SHETA* (28.6%; *n* = 14), followed by *ExhC* (16.3%; *n* = 8), *ExhB* (12.2%; *n* = 6), and both *ExhA* and *ExhD* (10.2%; *n* = 5). Only one isolate (2.0%) carried *SHETB* (Figure 1A; Figure 2A). When assessing the presence of multiple VFs within the same isolate, we observed that 24.5% (*n* = 12) of the isolates contained only one VF gene, 20.4% (*n* = 10) harbored two VFs, and two isolates (4.1%) carried three and four VF genes, respectively. Notably, 51.0% of isolates (*n* = 25) were negative for all evaluated VFs.

Regarding specific VF combinations, we identified nine different patterns, with two of them involving three or four VFs (Supplementary Table S1). The most common pattern was the association of *ExhC*-*SHETA* (12.2%; *p* < 0.01), followed by the combinations of *ExhB*-*SHETA* and *ExhD*-*SHETA*, each at 4.1% (*n* = 2). Notably, the only isolate harboring four VFs carried the combination *ExhA*-*ExhC*-*ExhD*-*SHETB*.

The analysis of *S. hyicus* isolates based on their VFs revealed that the first two dimensions of the PCA captured 65.7% of the total variability (Figure 1B). Dimension 1 accounted for 40.7% of the variability, primarily influenced by *SHETA* (68.0%) and *ExhC* (29.8%). In contrast, Dimension 2 explained 25.0% of the variability, with significant contributions from *ExhA* (33.7%), and *ExhB* (20.3%). Additionally, when evaluating the effect of AMR susceptibility profiling, we observed that the AMR to aminoglycosides accounted for 14.9% of the observe variability among *S. hyicus* isolates (PERMANOVA, *p* < 0.01), with most isolates non-wild type (NWT, resistant) to aminoglycosides being positive to both *SHETA* and *ExhC* (Figure 2A). There was no discernible impact from other antimicrobial classes, clinical signs, type of sample, production phase, pig breed or geographic origin.

### 2.3. Antimicrobial Susceptibility Profiling

Among the *S. hyicus* isolates evaluated in this study, 93.9% (*n* = 46) were resistant to at least one antimicrobial class, with 83.7% (*n* = 41) classified as multidrug resistant (MDR). Most of the isolates showed resistance to six (22.4%; *n* = 11) or seven (20.4%; *n* = 10) antimicrobial classes, with four isolates resistant to eight classes. The AMR patterns exhibited considerable heterogeneity, with 29 different combinations identified, none of which were predominant (Supplementary Table S2). The most common pattern was resistance to phenicols–pleuromutilins–tetracyclines–penicillins–aminoglycosides–macrolides–lincosamides (12.2%; *n* = 6), followed by phenicols–pleuromutilins–tetracyclines–penicillins–quinolones–macrolides–lincosamides and pleuromutilins–tetracyclines–penicillins–lincosamides (8.2%; *n* = 4).

Minimum inhibitory concentration (MIC) values for all evaluated antimicrobials are shown in Table 2. We observed that the NWT phenotype exceeded 50% for 9 of the 18 antimicrobials tested. NWT was particularly relevant for clindamycin (83.7%), tiamulin (81.6%), penicillin (75.5%), and chlortetracycline (73.5%). In contrast, the NWT phenotype was below 10% for ceftiofur and trimethoprim–sulfamethoxazole (4.1%).

Due to the absence of the specific antimicrobial concentrations that include the epidemiological cut-off (ECOFF) values for neomycin and spectinomycin on the microdilution plates used (Table 2), conclusive results for these antibiotics could not be obtained. For neomycin, seven isolates had MICs above the breakpoint, confirming the NWT phenotype, but it was not possible to determine the resistance status for the remaining 42 isolates. Conversely, for spectinomycin, 14 isolates had MICs below the breakpoint, indicating WT, but the resistance status for the remaining 35 isolates could not be verified.

AMR clustering at the class level demonstrated three main clusters (Figure 2A). The first one included those isolates with no or very few resistances. The other two included MDR *S. hyicus*, which were mainly divided by macrolide and, to a lesser extent, phenicol resistance. Notably, most aminoglycoside resistant *S. hyicus* exhibited a very similar MDR pattern and were mainly recovered from weaning piglets with epidermitis. It is remarkable that all *S. hyicus* isolates resistant to macrolides (59.1%; *n* = 29) were resistant to all evaluated members of this class, including tylosin, tilmicosin, and tulathromycin. This consistency was also observed for tetracyclines (NWT, 73.5%; *n* = 36), with only three isolates being wild type (WT, susceptible) for oxytetracycline but NWT to chlortetracycline. For aminoglycosides, despite notable differences among NWT phenotypes for gentamicin, neomycin and spectinomycin, no conclusive results could be drawn for the latter two due to the limitations in the antimicrobial concentration ranges, as discussed in the previous paragraph.

Principal component analysis (PCA) of *S. hyicus* isolates based on their AMR phenotype at the class level showed that the first two dimensions accounted for 57.5% of the variability. Dimension 1 represented 40.3% of the variability, mainly determined by macrolides (26.0%) and phenicols (21.9%). Dimension 2 accounted for 17.2% of the variability, predominantly determined by quinolones (66.8%) and aminoglycosides (17.3%). Further ordination analyses revealed that the type of sample, along with *ExhC* and *ExhD* VFs, collectively explained 26.7% of the AMR variability (PERMANOVA, *p* < 0.05). Specifically, the type of sample accounted for 13.2% (*p* < 0.01) (Figure 2B), showing similar AMR patterns in isolates from reproductive exudates and skin when compared to joints and brain. Within VFs, *ExhD* contributed 8.1% (*p* < 0.01) and *ExhC* contributed 5.4% (*p* < 0.05) to the variability. Although the clinical signs and the production phase also showed significant effects (PERMANOVA, *p* < 0.05), these were omitted due to the close association with the type of sample.

**Table 2:** Minimum inhibitory concentrations (MICs) of 18 antimicrobials against 49 *Staphylococcus hyicus* isolates from swine farms. The thick line represents the ECOFF used for each antimicrobial to classify isolates into wild type (WT) and non-wild type (NWT). Areas in gray represent values outside the concentrations included in the broth microdilution method.

Antimicrobial	Nº of Isolates with MIC (µg/mL)													MIC		WT		NWT	
	0.12	0.25	0.5	1	2	4	8	16	32	64	128	256	512	MIC_50_	MIC_90_	*n*	%	*n*	%
^c^ Penicillin	12	3	6	2	2	1	2	23						4	>8	12	24.5	37	75.5
^b^ Ampicillin		17	7	7	6	8	3	0	1					1	4	17	39.7	32	30.3
^c^ Ceftiofur		2	11	31	3	1	1							1	2	47	95.9	2	4.1
^b^ Gentamicin				38	1	1	2	3	4					≤1	16	39	79.6	10	20.4
^c^ Spectinomycin							0	0	5	9	35			>64	>64	14	28.6	-	-
^b^ Neomycin						42	6	0	1					≤4	8	-	-	7	14.3
^b^ Tulathromycin				1	8	10	2	1	0	0	27			>64	>64	21	42.9	28	51.1
^b^ Tilmicosin						20	0	0	1	0	28			≤4	≤4	20	40.8	29	59.2
^b^ Tylosin			2	10	8	0	0	0	1	28				>32	>32	20	40.8	29	59.2
^b^ Chlortetracycline			6	7	3	0	1	32						>8	>8	13	26.5	36	73.5
^b^ Oxytetracycline			12	4	0	0	0	33						>8	>8	16	32.7	33	67.3
^d^ Danofloxacin	7	23	2	3	14									0.25	>1	-	-	-	-
^b^ Enrofloxacin	19	10	2	3	2	13								0.25	>2	29	59.2	20	40.8
^c^ Clindamycin		8	0	2	0	2	3	0	36					>16	>16	8	16.3	41	83.7
^a^ SXT					47	2								≤2/38	≤2/38	47	95.9	2	4.1
^d^ Sulfadimethoxine												43	6	≤256	>256	-	-	-	-
^b^ Tiamulin			1	7	1	1	0	1	1	37				>32	>32	9	18.4	40	81.6
^b^ Florfenicol		0	0	0	8	17	15	9						8	>8	25	51	24	49

^a^ SXT: sulfamethoxazole–trimethoprim. ^b^ ECOFF defined by the EUCAST for *Staphylococcus hyicus*. ^c^ ECOFF defined by the EUCAST for *Staphylococcus aureus*. ^d^ ECOFF not defined.

### 2.4. Cooccurrence among Virulence Factors and Antimicrobial Susceptibility Profiling

**Figure 3:**
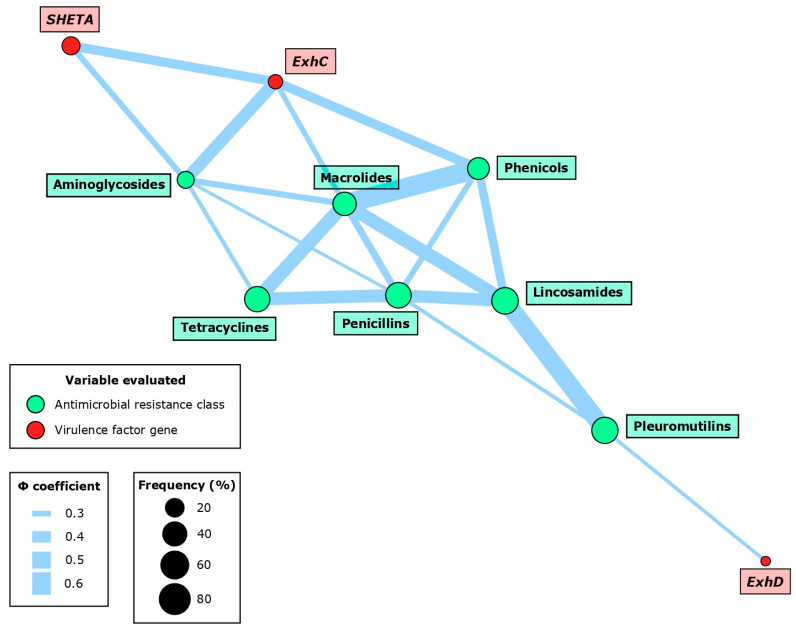
Network associations between virulence factor (VF) genes and antimicrobial resistance (AMR) at the class level in 49 *Staphylococcus hyicus* isolates from Spanish swine farms. Node size is determined by the percentage occurrence of the VF gene or AMR class. Edge size is proportional to the magnitude of the association based on the Φ coefficient. The network was constructed using significant associations (*p* < 0.05).

Several associations were observed between the VFs and AMR phenotypes at the AMR class level (Figure 3, Table 3). The most frequent interaction was the very strong co-occurrence of *ExhC* with three AMR classes: aminoglycosides (*p* < 0.01; Φ = 0.45), phenicols (*p* < 0.01; Φ = 0.40), and macrolides (*p* < 0.05; Φ = 0.31). Additionally, there was a significant association between *SHETA* and aminoglycosides (*p* < 0.05; Φ = 0.32), and between *ExhD* and pleuromutilins (*p* < 0.05; Φ = 0.28).

Within the VFs, we only detected the previously mentioned interaction between *ExhC* and *SHETA* (*p* < 0.01; Φ = 0.39). Among the AMR classes, 14 different associations were described involving seven different AMR classes: aminoglycosides, phenicols, macrolides, tetracyclines, penicillins, lincosamides, and pleuromutilins. Notably, there was a very strong co-occurrence between antimicrobials that share AMR mechanisms, such as macrolides with phenicols (*p* < 0.001; Φ = 0.61) and lincosamides (*p* < 0.001; Φ = 0.48), or the latter also with pleuromutilins (*p* < 0.001; Φ = 0.57).

### 2.5. Biofilm Formation

**Figure 4:**
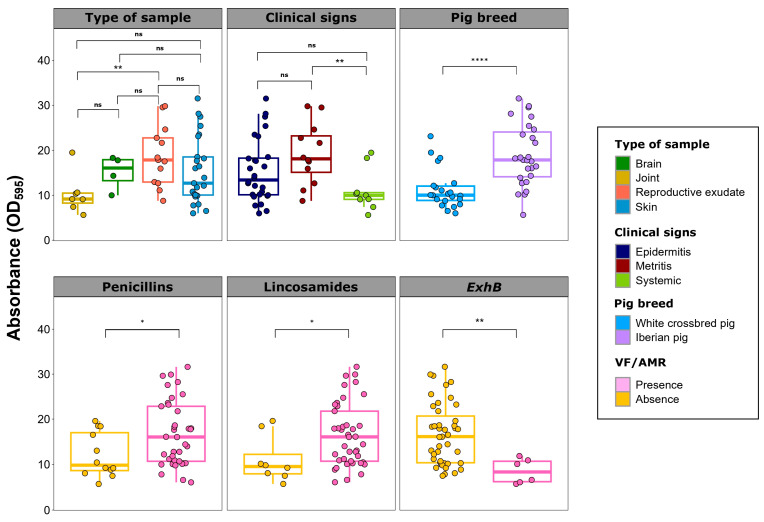
Biofilm formation of *Staphylococcus hyicus* isolates from Spanish swine farms. Boxplots illustrating the quantitative biofilm formation of *S. hyicus*, comparing the type of sample and the clinical signs of the animals, the pig breed, the antimicrobial resistance to penicillins and lincosamides, or the presence of the *ExhB* virulence factor (VF) gene. Quantification was performed as the difference from the negative control (DC) in absorbance (OD_595_). Each *S. hyicus* isolate is represented by a dot with a horizontal jitter for visibility. The horizontal box lines represent the first quartile, the median, and the third quartile. Whiskers extend to 1.5 times the interquartile range. Differences between groups were evaluated using the Wilcoxon rank-sum test. The level of statistical significance was represented with asterisks: four asterisks (****) indicated a *p*-value less than 0.0001; two asterisks (**) indicated a *p*-value between 0.001 and 0.01; one asterisk (*) indicated a *p*-value between 0.01 and 0.05; non-significance (ns) indicated a *p*-value higher than 0.05.

All evaluated *S. hyicus* isolates exhibited a very high biofilm formation capacity (DC = 15.6 ± 7.0), with wide differences that ranged from 6.6 to 31.6. Significant differences in biofilm formation were observed in relation to metadata, VFs, and AMR phenotypes (Figure 4).

Isolates from animal with metritis showed significantly higher biofilm formation than those from piglets with systemic clinical signs (*p* < 0.01). This is reflected in lower biofilm formation in isolates from joints compared to those from reproductive exudates (*p* < 0.01). Regardless of the region, isolates from Iberian pigs had higher biofilm formation than those from white crossbred pigs (*p* < 0.001). Regarding VFs, isolates harboring *ExhB* showed lower biofilm formation capacity than those without this VF gene (*p* < 0.01). Conversely, resistance to penicillins and lincosamides was consistently associated with significantly higher biofilm formation (*p* < 0.05).

## 3. Discussion

Current limitations on antimicrobial use in the European Union [13] have led to increasing concerns about the potential reemergence of pathogens in swine production, such as *S. hyicus*. The lack of updated information on this pathogen, particularly in Europe, makes it necessary to further characterize clinical *S. hyicus* isolates. Here, by establishing associations among pathogenicity markers, biofilm formation capacity, and AMR patterns, we enhanced the understanding and management of clinical *S. hyicus* recovered from Spanish swine farms, contributing to more targeted and effective therapeutic strategies.

*S. hyicus* is the primary causative agent of EE, particularly in suckling and weaning piglets [2]. In this study, over half of the isolates were recovered from animals exhibiting clinical signs of epidermitis. Additionally, we isolated *S. hyicus* from sows and boars primarily affected by reproductive signs, particularly sows with metritis. Although not extensively studied, prior research has reported the potential role of *S. hyicus* in reproductive diseases in sows, especially inducing mastitis and metritis [4], as observed in this study. About 25% of clinical cases were associated with systemic infections, with isolates recovered from joints and the brain. While not frequently described, a previous study reported the involvement of *S. hyicus* in causing arthritis in suckling to weaning piglets [4] and suppurative pneumonia and pericarditis in piglets [15]. However, none have characterized its presence in brain samples. This systemic dissemination may be explained by the bacteriemia caused by hematogenous spread or external inoculation following skin wounds [16]. Indeed, this has been previously reported in systemic infection in humans with endocarditis [17] and spondylodiscitis [16]. These findings demonstrate that *S. hyicus* should be considered not only as a cutaneous pathogen but also as a potential systemic agent primarily affecting piglets.

The characterization of the pathogenicity of *S. hyicus* requires evaluating the presence of exfoliative toxins, particularly in toxigenic strains causing EE. In this study, nearly half of the isolates (49.0%) were positive for one or more of the six characterized toxins, consistent with previous findings [18]. Although previous studies have examined the prevalence of these toxins [14,19,20], it may be challenging to provide a detailed comparative analysis due to geographical differences and the potential changes in prevalence over time. We observed that *SHETA* was the most frequently detected VF gene (28.6%). SHETA is a toxigenic VF that has been described to contribute to the clinical signs of EE, although its toxigenic mechanism remains unknown [19]. In contrast to the plasmid location of the *SHETB* gene, *SHETA* is chromosomally encoded [9] and genetically related to the *ExhB* gene, with 98.6% DNA homology [21]. Additionally, we observed a significant association of *SHETA* with *ExhC,* a gene encoding one the four Exh toxins, which was the second most prevalent VF (16.3%) in this study. Exh toxins, encoded by the *ExhA*, *ExhB*, *ExhC* and *ExhD* genes, are characteristic of toxigenic *S. hyicus* involved in EE. These toxins, which have been detected at a low frequency in this study, cause a loss of cell adhesion in the epidermis of porcine skin by cleaving desmoglein-1 [22]. Therefore, evaluating exfoliative toxins may be essential for identifying and studying *S. hyicus* isolates from clinical cases, especially EE. However, more than half of the isolates (51.0%) were negative for the six characterized toxins, regardless of the sample type or the clinical signs. This highlights the need for further characterization of other potential pathogenic mechanisms in *S. hyicus*.

Biofilm formation has been identified as an essential pathogenic mechanism in related bacteria, such as *S. aureus* and coagulase-negative staphylococci [11]. However, until now, to the best of our knowledge, no previous study has specifically addressed biofilm formation in *S. hyicus* and its underlying mechanisms. Here, we demonstrate for the first time the robust in vitro biofilm formation of *S. hyicus*, regardless of sample type. This suggests a high survival capacity in certain environments, which could contribute to the persistence of other bacterial pathogens in coinfection processes. Notably, a significantly lower biofilm formation was observed among *S. hyicus* from systemic locations compared to those from reproductive exudates. Previous studies have shown the importance of biofilm formation for survival in reproductive environments, such as *S. aureus* in bovine mastitis [23] or *Trueperella pyogenes* in cattle endometritis [24]. The lower biofilm formation in joint-related *S. hyicus* contrasts with previous studies suggesting strong biofilm aggregates in synovial fluid caused by *S. aureus* [25] or *Staphylococcus lugdunensis* [26]. However, it should be noted that, despite the significantly lower biofilm formation, the levels are still exceptionally high compared to other porcine pathogens [27]. Additionally, there is an association between biofilm formation and resistance to certain antimicrobials, such as penicillins and lincosamides. A recent study reported the association between biofilm formation and AMR in *S. aureus* [28], highlighting the complexity and significance of biofilm formation not only in pathogenicity but also in therapeutic success and the survival of *S. hyicus* in its biological niche.

Antimicrobials, along with biosecurity and management practices such as optimizing animal density, nutrition, hygiene, and measures aimed at reducing skin injuries in piglets, remain essential for controlling *S. hyicus* outbreaks, particularly in the absence of a commercial vaccine [29]. However, antimicrobial use should be guided by responsible stewardship principles to limit the development and spread of AMR. Therefore, evaluating the phenotypic AMR profile of *S. hyicus* recovered from clinical samples is crucial. Here, we observed a remarkably high prevalence of MDR *S. hyicus* (83.7%) on Spanish swine farms, consistent with previous studies from Japan and Brazil [14,30]. Notably, 51% of *S. hyicus* were resistant to six to eight different antimicrobial classes, highlighting the particularly high AMR observed in most isolates. When evaluating AMR by specific antimicrobials, we found the highest AMR frequencies against those most frequently used in Spanish swine production [31], such as lincosamides (83.7%), pleuromutilins (81.6%), penicillins (75.5%), and tetracyclines (73.5%). Resistance to last-resort antimicrobials was remarkably lower, particularly for ceftiofur (4.1%) and, to a lesser extent, for enrofloxacin (40.8%). This contrasts with higher enrofloxacin resistance reported in Brazil [14], likely due to less restrictive legislation on antimicrobial use in food-producing animals. These findings underscore the impact of antimicrobial use on the AMR phenotype of *S. hyicus* and emphasize the need for targeted diagnosis to promote responsible antimicrobial use and therapeutic success.

Several factors influence the selection of specific bacterial strains, with antibiotic use being one of the primary factors [32]. AMR selective pressure can also lead to cross-selection and co-selection events. Cross-selection occurs when a single antimicrobial resistance gene (ARG) confers resistance to multiple antimicrobial classes, while co-selection occurs when two genes are physically linked on a piece of DNA so are inherited together, either chromosomally encoded or located in potentially mobilizable genomic regions [33]. In this study, we observed strong associations between certain antimicrobial classes, such as lincosamides with pleuromutilins (*p* < 0.001; Φ = 0.57) or macrolides (*p* < 0.001; Φ = 0.48). This may be explained by the cross-resistance conferred by certain ARGs such as *lsa(E)* or *erm(T)*, previously reported in *S. hyicus* isolates from Spanish swine farms [34], which confer resistance to lincosamides and pleuromutilins, and macrolides and lincosamides, respectively. The co-selection of certain AMR phenotypes with *ExhC* and *ExhD* may be due to the possible association of these VFs and ARGs in certain potentially mobile genomic regions. Despite extensive characterization [35], little is known about the genomic location of these *Exh* genes. Furthermore, we observed a significant association between the AMR phenotype and the sample type, indicating potential adaptations of certain pathogenic *S. hyicus* strains or lineages to specific biological niches, as has been reported for *S. aureus* due to its genomic plasticity [36]. This is demonstrated by the differential presence of the chromosomally encoded *SHETA*. Therefore, further genomic characterization of *S. hyicus* is needed to clearly confirm the associations observed in this study.

In conclusion, here we provide significant insight into the wide diversity and complexity of virulence, AMR, and biofilm formation in *S. hyicus* isolates from Spanish swine farms, recovered from animals with cutaneous, reproductive and systemic clinical signs. A high prevalence of MDR isolates was observed, with particularly high resistance to antimicrobials commonly used in Spanish swine production. Additionally, only about half of the isolates harbored at least one VF gene, despite their isolation from clinical cases, suggesting the presence of other virulence mechanisms. Notably, all isolates demonstrated robust biofilm formation capacity, indicating a potential for survival and persistence in various biological niches. The associations between virulence factors, biofilm formation, and AMR patterns indicate the possible mechanisms of adaptation and selection in *S. hyicus*. These findings underscore the need for targeted diagnostics and further genomic characterization to improve the management and therapeutic strategies against *S. hyicus* in swine production.

## 4. Materials and Methods

### 4.1. Sampling and Bacterial Isolation and Characterization

Clinical samples were collected from the skin, reproductive exudates, joints, and brain of pigs displaying clinical signs compatible with epidermitis, metritis and systemic clinical signs such as polyserositis. These samples were obtained from both Iberian and white crossbred pigs from Spanish swine farms, with collections occurring from May to December 2023.

The collected swabs were cultured on blood agar plates (Oxoid, Madrid, Spain) for 24 h at 37 °C under aerobic conditions. Presumptive white, non-hemolytic colonies were subsequently subcultured on tryptic soy agar (TSA) (Condalab, Madrid, Spain) and incubated for 24 h at 37 °C. These colonies were confirmed as *S. hyicus* using MALDI-TOF mass spectrometry employing the IVD MALDI Biotyper (Bruker Daltonik, Bremen, Germany) according to the manufacturer’s standard protocols.

### 4.2. Molecular Characterization of Virulence Factors

For molecular characterization of the VFs, DNA extraction of *S. hyicus* isolates was performed. A single colony was inoculated in 100 µL sterile distilled water and boiled for 10 min at 100 °C. Subsequently, it was centrifuged at 12,000 rpm for 10 min and the supernatant with the DNA extracted was transferred to a new sterile microtube for further analyses. The purified supernatant was stored at −20 °C until further use.

The molecular characterization of the VSs was performed using simplex PCRs for the *SHETA* and *SHETB* genes and a multiplex PCR for the *ExhA*, *ExhB*, *ExhC*, and *ExhD* genes. The primers (20 µM) and the amplicon size (in base pairs, bp) are available in Supplementary Table S3.

The amplification protocol for the *SHETA* simplex PCR included a denaturization step at 94 °C for 3 min, followed by 30 amplification cycles that included a 30 s denaturization at 94 °C, 30 s, annealing at 58 °C, and a 1 min and 10 s extension at 72 °C. It finalized with a final extension at 72 °C for 10 min. The *SHETB* simplex PCR consisted of a denaturization step at 94 °C for 2 min, followed by 35 amplification cycles that included a 30 s denaturization at 94 °C, 30 s, annealing at 50 °C, and a 1 min and 10 s extension at 72 °C. It finalized with a final extension at 72 °C for 10 min.

For its part, the multiplex PCR included a denaturization step at 94 °C for 2 min, followed by 35 amplification cycles with a 30 s denaturization at 94 °C, 30 s annealing at 50 °C, and a 1 min and 10 s extension at 72 °C. It finalized with a final extension at 72 °C for 10 min.

The mixture for both multiplex and simplex PCR reactions consisted of 3 μL of extracted DNA, 0.5 μL of DNA polymerase (5 U/μL) (Biotools, Madrid, Spain), 6.5 μL of 10X amplification PCR buffer with MgCl_2_ (Biotools, Madrid, Spain), 0.5 μL of each primer (20 μM) (Roche Diagnostics, Basel, Switzerland), 1 μL of dNTPs (25 mM each) (Biotools, Madrid, Spain), and nuclease-free water (Invitrogen, Carlsbad, CA, USA) to reach a final volume of 50 μL.

### 4.3. Antimicrobial Susceptibility Testing

Antimicrobial susceptibility testing followed the procedures outlined by the European Committee on Antimicrobial Susceptibility Testing (EUCAST) [37]. The MIC of the tested antimicrobials was determined using the broth microdilution method. It established the MIC_50_ and MIC_90_, which are the concentrations that inhibit the growth of 50% and 90% of the isolates, respectively. The microbiological resistance was determined in accordance with the ECOFF value, thus dividing the microorganisms depending on whether they have (non-wild type, NWT) or not (wild type, WT) acquired resistance mechanisms to each antimicrobial [38]. Non-wild type and resistant phenotype are indistinctly used throughout this study. MDR was defined as acquired non-susceptibility to at least one agent in three or more antimicrobial classes [39]. A microorganism susceptible to all antimicrobials tested was defined as pansusceptible (PNS).

AMR was evaluated with BOPO6F Sensititre plates (TREK Diagnostic Systems, East Grinstead, UK). The antimicrobials evaluated and their ECOFFs are shown in Table 2. *Staphylococcus aureus* ATCC 29213 was used as the control strain. The ECOFFs were primarily selected for *S. hyicus* and if no information was available, they were extrapolated from *S. aureus* (e.g., penicillin, ceftiofur, spectinomycin, and clindamycin). No ECOFF could be established for sulfadimethoxine and danofloxacin.

*S. hyicus* isolates were cultured on TSA at 37 °C for 24 h under aerophilic conditions. After its growth, a single colony was resuspended in 5 mL of 0.9% sterile saline solution to reach a turbidity of McFarland 0.5. Fifty microliters of the bacterial suspension were transferred to 11 mL of Mueller-Hinton broth (TREK Diagnostic Systems, East Grinstead, UK) and 50 µL per well was dispensed with the Sensititre AIM Automated Inoculation Delivery System (TREK Diagnostic Systems, East Grinstead, UK). Plates were sealed and incubated in an aerophilic atmosphere at 37 °C for 24 h.

### 4.4. Biofilm Formation Assay

Biofilm formation of *S. hyicus* was quantified by crystal violet staining, following a biofilm formation protocol previously described for *S. aureus* [40] with slight modifications. Briefly, a single colony was inoculated into 96-well polystyrene microfiber cell culture-treated plates (Corning Incorporated, Corning, NY, USA) containing 200 µL of tryptic soy broth (TSB) (Condalab, Madrid, Spain) supplemented with 0.1% glucose (VWR, Leuven, Belgium) and incubated for 24 h at 37 °C under aerobic conditions. Sterile TSB was used as the negative control.

Following incubation, the culture medium and unattached bacteria were removed by aspiration. The formed biofilms were stained with 100 μL of 2% crystal violet for 30 min, washed three times with distilled water, and dried at 37 °C for 15 min. To release the dye, 100 μL of 95% ethanol was added and the plates were briefly agitated. The absorbance of the biofilm biomass was quantified at 595 nm (A595). All assays were conducted in triplicate to ensure reliability of the results. The final optical density (OD) value of each isolate was expressed as the mean of the three measurements subtracting the average OD of the negative control (difference from the control, DC), to lessen the possible unevenness in absorbance quantification.

### 4.5. Data Analysis and Results Visualization

A database was created in an Excel sheet (Microsoft Office 365) to include metadata, VFs (presence or absence), antimicrobials tested (WT or NWT) and biofilm formation. The metadata were the Spanish region from which the clinical sample was recovered, clinical signs (epidermitis, metritis, and systemic), type of sample (skin, joint, brain or reproductive exudate), production phase (suckling, weaning, and sow/boar), and pig breed (Iberian breed or white crossbred pig). AMR analyses were performed at the antimicrobial and antimicrobial class level. Biofilm formation was expressed numerically as DC and categorized based on the DC value into low (DC ≤ 2), medium (2 > DC ≤ 3), and high (DC > 3), as previously described [41]. All analyses were conducted using R version 4.3.2 (31 October 2023 ucrt) [42]. Plots were produced using the *ggplot2* version 3.5.1 [43], *igraph* version 2.0.3 [44] and *ggraph* version 2.2.1 [45] packages and further modified using the software Inkscape version 1.3.2 (https://inkscape.org/, accessed on 11 July 2024).

A clustering of *S. hyicus* isolates was performed according to their AMR phenotype using the unweighted pair group method with arithmetic mean (UPGMA) as the hierarchical clustering method. The *pheatmap* package [46] was used for the representation of the clustered heatmaps of isolates. Comparisons among isolates for metadata, VFs, and AMR phenotype were carried out with Fisher’s exact test. Comparisons for DC biofilm formation was carried out using the Wilcoxon rank-sum test. *p*-values were adjusted following the Benjamini and Hochberg method [47]. Significance was established at *p* < 0.05.

Ordination of *S. hyicus* isolates based on their AMR phenotype and VFs was estimated using a Jaccard distance matrix and analyzed by principal component analysis (PCA), and the two main dimensions for the principal components were characterized. The effect of the metadata, AMR phenotypes, and VFs was determined by permutational multivariate analysis of variance (PERMANOVA) using distance matrices with adonis2 function.

Associations between VF genes and the AMR phenotype consisted of a primary approach involving the execution of Fisher’s exact test to identify significant associations between the VFs and AMR profiles. It was further complemented by calculation of the Phi coefficient (Φ) to measure the strength of these associations. Subsequently, the percentage of occurrence for each VF and antimicrobial was computed. Significant associations were visualized in a network graph, where node sizes were determined by their percentage occurrence and edge sizes reflected the magnitude of Φ. Φ was categorized as very strong (Φ > 0.25), strong (Φ > 0.15), moderate (Φ > 0.10) or weak (Φ > 0.05).

## Figures and Tables

**Table 1:** Characteristics of the 49 *Staphylococcus hyicus* recovered from Spanish swine farms.

		Frequence (*n*)	Percentage (%)
**Geographic origin**	Castile and León	9	18.4
	Catalonia	10	20.4
	Extremadura	11	22.4
	Galicia	6	12.2
	Region of Murcia	13	26.5
**Type of pig**	Iberian pig	27	55.1
	White crossbred pigs	22	44.9
**Production phase**	Suckling	12	24.5
	Weaning	24	49.0
	Sow/Boar	13	26.5
**Type of sample**	Brain	7	14.3
	Joint	4	8.2
	Reproductive exudate	13	26.5
	Skin	25	51.0
**Clinical signs**	Epidermitis	28	57.1
	Metritis	12	24.5
	Systemic	9	18.4

**Table 3:** Significant associations (*p* < 0.05) between virulence factors and phenotypic antimicrobial resistance (AMR) at the class level in 49 *Staphylococcus hyicus* isolates from Spanish swine farms.

Pairwise Association	Φ Coefficient	Φ Categorization	*p*-Value
Aminoglycosides—*ExhC*	0.45	Very strong	0.001
Phenicols—*ExhC*	0.40	Very strong	0.002
Aminoglycosides—*SHETA*	0.32	Very strong	0.023
Macrolides—*ExhC*	0.31	Very strong	0.015
Pleuromutilins—*ExhD*	0.28	Very strong	0.037
*SHETA—ExhC*	0.39	Very strong	0.004
Macrolides—Phenicols	0.61	Very strong	<0.001
Lincosamides—Pleuromutilins	0.57	Very strong	<0.001
Macrolides—Tetracyclines	0.49	Very strong	0.001
Lincosamides—Macrolides	0.48	Very strong	<0.001
Penicillins—Tetracyclines	0.46	Very strong	0.001
Lincosamides—Penicillins	0.45	Very strong	0.001
Lincosamides—Phenicols	0.38	Very strong	0.004
Macrolides—Penicillins	0.35	Very strong	0.008
Macrolides—Aminoglycosides	0.33	Very strong	0.016
Penicillins—Phenicols	0.32	Very strong	0.018
Lincosamides—Tetracyclines	0.30	Very strong	0.023
Aminoglycosides—Tetracyclines	0.29	Very strong	0.021
Penicillins—Pleuromutilins	0.28	Very strong	0.029
Aminoglycosides—Penicillins	0.27	Very strong	0.024

## Data Availability

The original contributions presented in the study are included in the article/Supplementary Materials; further inquiries can be directed to the corresponding author/s.

## Supplementary Materials

The following supporting information can be downloaded at: https://www.mdpi.com/article/10.3390/antibiotics13090871/s1, Table S1: Combination of virulence factor genes in 49 *Staphylococcus hyicus* isolates from Spanish swine farms; Table S2: Combination of phenotypic antimicrobial resistance at the class level in 49 *Staphylococcus hyicus* isolates from Spanish swine farms; Table S3: Primers used for molecular characterization of *Staphylococcus hyicus* virulence factors.
