# Supplementary material for: Insights into the Virulence and Antimicrobial Resistance of Staphylococcus hyicus Isolates from Spanish Swine Farms
Source: Antibiotics (Basel). 2024 Sep 11;13(9):871. doi: 10.3390/antibiotics13090871 (PMC11428503; doi:10.3390/antibiotics13090871)
Supplement: Supplementary file 1 [file antibiotics-13-00871-s001.zip › Table S3.docx]

**Supplementary Table S3.** Primers used for molecular characterization of S. hyicus virulence factors.

| **Gene** | **Primer sequences** | **Amplicon size (bp)** | **Reference** |
| --- | --- | --- | --- |
| ***ExhA*** | **ExhA-F:5’-CTCAGAGGGCAAAGTAGAATTGCCT-3’**  **ExhA-R:5-AGGATTTCTGTGCCATCATCAAATC-3’** | **389** | **[21]** |
| ***ExhB*** | **ExhB-F: 5’AGAGAATGTATCACATGCAAGCGAA 3’**  **ExhB-R: 5’-CTCTAGTGGTTTTACAATGTCACCT-3’** | **444** | **[21]** |
| ***ExhC*** | **ExhC- F: 5’CTTAGCAGACGAAGAAAGTGACTTG 3’**  **ExhC-R: 5’-ACCTGTTGCTACAGCTTCTCCTTGA-3’** | **183** | **[21]** |
| ***ExhD*** | **ExhD-F: 5’-AGAATTGGTGATCCTGAGTTACCAC-3’**  **ExhD-R: 5’-TTTGGAGCAGGTTTAGAATTAGCGA-3’** | **259** | **[21]** |
| ***SHETA*** | **SHETA-F: 5’-GAACACGTTTTTCAGCCATATCTCC-3’**  **SHETA-R: 5’-CGATTACAGTTGCCAATACCGTTTC-3’** | **615** | **[19]** |
| ***SHETB*** | **SHETB-F: 5’-CATTTGCACAATTTCAGTCCCTATG-3’**  **SHETB-R: 5’-AACATGGATTCCAACTAGTTCACCA-3’** | **672** | **[21]** |
